# Complete Mitochondrial Genome of *Eruca sativa* Mill. (Garden Rocket)

**DOI:** 10.1371/journal.pone.0105748

**Published:** 2014-08-26

**Authors:** Yankun Wang, Pu Chu, Qing Yang, Shengxin Chang, Jianmei Chen, Maolong Hu, Rongzhan Guan

**Affiliations:** 1 State Key Laboratory of Crop Genetics and Germplasm Enhancement, Nanjing Agricultural University, Nanjing, Jiangsu, China; 2 Institute of Economic Crop, Jiangsu Academy of Agricultural Sciences, Nanjing, Jiangsu, China; 3 Nanjing Agricultural University, Jiangsu Collaborative Innovation Center for Modern Crop Production, Nanjing, Jiangsu, China; Zhejiang University, China

## Abstract

*Eruca sativa* (Cruciferae family) is an ancient crop of great economic and agronomic importance. Here, the complete mitochondrial genome of *Eruca sativa* was sequenced and annotated. The circular molecule is 247 696 bp long, with a G+C content of 45.07%, containing 33 protein-coding genes, three rRNA genes, and 18 tRNA genes. The *Eruca sativa* mitochondrial genome may be divided into six master circles and four subgenomic molecules via three pairwise large repeats, resulting in a more dynamic structure of the *Eruca sativa* mtDNA compared with other cruciferous mitotypes. Comparison with the *Brassica napus* MtDNA revealed that most of the genes with known function are conserved between these two mitotypes except for the *ccmFN2* and *rrn18* genes, and 27 point mutations were scattered in the 14 protein-coding genes. Evolutionary relationships analysis suggested that *Eruca sativa* is more closely related to the *Brassica* species and to *Raphanus sativus* than to *Arabidopsis thaliana*.

## Introduction

Mitochondria supply energy in the form of ATP through oxidative phosphorylation in almost all eukaryotic cells [1]. In comparison to their counterparts in animals and fungi, plant mitochondrial (mt) genomes have unique features, such as large and dramatic variations in size [2], dynamic structure [3], extremely low rate of point mutations [4] and incorporation of foreign DNA [5]. The largest known mitochondrial genomes are those of seed plants, with sizes ranging from 208 kb for *Brassica hirta* [6] to over 11.3 Mb for *Silene conica* [4]. The dramatic variation may occur within closely related species [7]. Active recombination via repeated sequences appear to be responsible for the dynamic nature and multipartite organization of the mt genome in all angiosperms investigated [8], which may produce significantly different gene orders even among close relatives [9].

Mitochondria play an important role in plant growth and development. Genomic rearrangements involving substoichiometric shifting (SSS), a consequence of intermediate repeat DNA exchange [10], is often accompanied by changes in the plant's phenotype. SSS activity in plant mitochondria has been reported to be associated with cytoplasmic male sterility [11], nitrate sensing and GA-mediated pathways for growth and flowering [12]. Plant mitochondria have also been associated with stress responses [13] and regulation of programmed cell death [14]. Therefore, determining mitochondrial genomes is important for determining specific metabolic activities of plants [15].

*Eruca sativa* Mill.or *Eruca vesicaria* subsp. *sativa* (Miller) (Garden rocket), a member of the Cruciferae family, has several desirable agronomic traits, such as resistance to salt, drought, white rust and aphids [16]–[18]. Introducing these beneficial genes of *E. sativa* into economically important cultivated species will promote crop improvement [19], [20]. Crosses of *E. sativa* with other species of the family Cruciferae, including *B. rapa*, *B. juncea*, and *B. oleracea*, have been reported [20].

To date, several mt genomes from the *Cruciferae* family have been sequenced, including *Arabidopsis thaliana* (*tha*) [21], *Raphanus sativus* (*sat*) [22] and five species from the *Brassica* genus, i.e., *B. napus* (*pol*, *nap*), *B. rapa* (*cam*), *B. oleracea* (*ole*), *B. juncea* (*jun*), and *B. carinata* (*car*) [23]–[25]. In this study, we reported the complete mitochondrial genome sequences of *E. sativa* and provide a comparison with other sequenced *cruciferous* mt genomes. This research will help to characterize the *E. sativa* crop and further our understanding of the evolution of mitochondrial genomes within the *Cruciferae* family.

## Materials and Methods

### Mitochondrial DNA isolation and sequencing

A commercial cultivar of *E. sativa* was used in this study. Mitochondrial DNA was isolated from 7-day-old etiolated seedlings according to Chen's methods (Chen et al., 2011), and stored at −80°C until use. Genome sequencing was performed using the GS-FLX platform (Roche, Branford, CT, USA). The reads were assembled into contigs using Newbler v.2.6. Sanger sequencing of PCR products was used to join the contigs to form the complete genome.

### Sequence data analysis

The NCBI database (http://www.ncbi.nlm.nih.gov/) was searched for mitochondrial sequences annotation, using previously annotated mitochondrial genes from angiosperms as query sequences. The tRNAs were identified using the tRNA scan-SE software (http://lowelab.ucsc.edu/tRNAscan-SE/). Putative open reading frames (ORFs) with a minimum size of 100 codons were predicted and annotated using ORF-Finder (http://www.ncbi.nlm.nih.gov/gorf/gorf.html). The circular map was drawn using OGDraw v1.2 (http://ogdraw.mpimp-golm.mpg.de/). Repeats analysis was performed as previously described [25].

### Comparing mitochondrial genomes and evolutionary analysis

The *E. sativa* mitochondrial genome sequence presented here was compared with eight other reported Cruciferae mitotypes: *B. rapa* (GenBank: NC_016125), *B. oleracea* (GenBank: NC_016118), *B. juncea* (GenBank: NC_016123), *B. carinata* (GenBank: NC_016120), *B. napus* (GenBank: NC_008285), *B. napus* cultivar Polima (EMBL: FR715249), *R. sativus* (GenBank: JQ083668) and *A. thaliana* (GenBank: NC_001284), using NCBI-blastn. For comparison, the exons of 32 protein coding genes (*atp1*, *atp4*, *atp6*, *atp8*, *atp9*, *ccmB*, *ccmC*, *ccmFc*, *ccmFN1*, *ccmFN2*, *cob*, *cox1*, *cox2-1*, *cox3*, *matR*, *nad1*, *nad2*, *nad3*, *nad4*, *nad4L*, *nad5*, *nad6*, *nad7*, *nad9*, *rpl2*, *rpl5*, *rpl16*, *rps3*, *rps4*, *rps7*, *rps12*, *tatC*), which were shared by these nine species, were extracted and sequentially joined together. A neighbor-joining tree [26] was constructed with MEGA 5, using the Kumar method [27]. The number of bootstrap replications was set as 1000 [28].

## Results

### The mitochondrial genome of *E*. *sativa*

**Figure 1:**
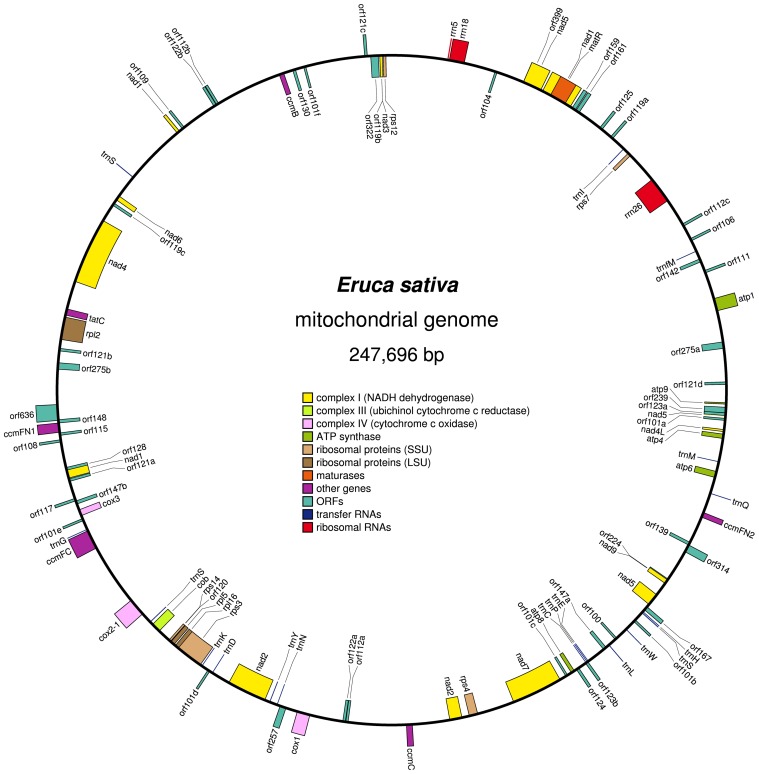
Mitochondrial genome map of *Eruca sativa.* Features on the clockwise- and counter-clockwise-transcribed strands are drawn on the inside and outside of the circle, respectively. The figure was drawn using OGDraw v1.2.

The mitochondrial genome of *E. sativa* was assembled as a single circular molecule of 247 696 bp (Figure 1, deposited in GenBank under the accession KF442616). The overall GC content of the mtDNA is 45.07%, which is comparable to those of other mtDNAs of *Cruciferae*. The largest part of the *E. sativa* mtDNA comprises the non-coding sequences (85.14%), which is slightly smaller than the average non-coding sequences content (89.4±3.1%) in other reported angiosperm mitochondrial genomes [29]. Genes account for 26.27% of the genome (65 070 bp in total length), 56.61% of which represent exons (36 837 bp) and 43.39% represent introns (28 233 bp).

### Gene content and ORFs

Using BLAST and tRNA scan-SE, 54 genes were identified, including 33 protein coding genes, three rRNA genes (5S, 18S and 26S rRNAs) and 18 transfer RNA genes (Figure 1, Table 1). The 33 protein coding genes (PCGs) were in the range of 225 bp (*atp9*) to 7 979 bp (*nad4*), including 18 genes for components of the electron transport chain and ATP synthase: nine subunits of complex I (*nad1-7*, *4L*, *9*), one subunit of complex III (*cob*), three subunits of complex IV (*cox1-3*) and five subunits of complex V (*atp1*, *4*, *6*, *8*, *9*). In addition, there are five genes for cytochrome c biogenesis (*ccmB*, *ccmC*, *ccmFN1/ccmFN2* and *ccmFC*), eight genes for ribosomal proteins (*rpl2*, *5*, *16* and *rps3*, *4*, *7*, *12*, *14*), two genes for maturase (*matR*) and one gene for other functions (*tatC*). The total length of the 33 PCGs of *E. sativa* mtDNA is 58 569 bp, accounting for 23.64% of its total mtDNA genome length, which is lower than that of the *Brassica* and *R. sativus* mitotypes. Nine genes had an exon–intron structure. All exons of *ccmFC* (exons a, b), *nad2* (a-e), *cox2* (a, b), *nad4* (a-d), *nad7* (a-e), *rps3* (a, b) and *rpl2* (a, b) were cis-spliced, whereas some exons of *nad1* and *nad5* were trans-spliced as follows: *nad1a*/*nad1b-e*; *nad5a*, *b*, *d*, *e*/*nad5c* (the slash indicating trans-spliced exons). ATG is the most commonly used initiation codon for mitochondrial PCGs in *E. sativa*, except for *nad1* (start with ACG), *matR* (start with AGA) and *tatC* (start with ATT), as predicted by previous studies (Handa, 2003). Ten genes (*nad4*, *cob*, *ccmC*, *ccmFN1/ccmFN2*, *cox3*, *atp8*, *atp9*, *rpl2* and *rps12*) are predicted to terminate with TGA and six (*atp1*, *nad7*, *rps3*, *rps14*, *matR* and *tatC*) with TAG; other PCGs use TAA as their termination codon.

**Table 1:** Gene content of the mitochondrial DNA of *Eruca sativa*.

Product group	Gene							
Complex I	*nad1*	*nad2*	*nad3*	*nad4*		*nad4L*		
	*nad5*	*nad6*	*nad7*	*nad9*				
Complex III	*cob*							
Complex IV	*cox1*	*cox2-1*	*cox3*					
Complex V	*atp1*	*atp4*	*atp6*	*atp8*		*atp9*		
Ribosome large subunit	*rpl2*	*rpl5*	*rpl16*					
Ribosome small subunit	*rps3*	*rps4*	*rps7*	*rps12*		*rps14*		
Cytochrome c biogenesis	*ccmB*	*ccmC*	*ccmFC*		*ccmFN1*		*ccmFN2*	
Intron maturase	*matR*							
Protein translocase	*tatC*							
rRNA genes	*rrn5*	*rrn18*	*rrn26*					
tRNA genes	*trnN*	*trnD*	*trnC*	*trnE*		*trnQ*		*trnG*
	*trnH*	*trnI*	*trnK*	*trnM*		*trnfM*		*trnP*
	*trnW*	*trnY*	*trnL*	*trnS*(3×)				

18 tRNA sequences (1 383 bp) were found in *E. sativa* mtDNA (Table 2), in the range of 71–88 bp in length. The A+T content of the tRNA genes is 48.81%, which is lower than the overall A+T composition of the mtDNA. Among these genes, tRNAs for 15 amino acids, including duplication of the methionine (Met) and triplication of the serine (Ser), are encoded. The genome lacks tRNAs for the amino acids alanine (Ala), valine (Val), phenylalanine (Phe), threonine (Thr) and arginine (Arg). To enable gene expression for protein synthesis in mitochondria, the missing tRNAs may be supplied by either the chloroplast or nuclear genomes [30].

**Table 2:** Recognition of anticodons by tRNA genes found in the mtDNA of *Eruca sativa.*

Name	Type	Anticodon	Length(bp)	Orientation
chloroplast origin				
*trnD*	Asp	GTC	74	inverted
*trnH*	His	GTG	74	direct
*trnL*	Leu	CAA	81	direct
*trnM*	Met	CAT	73	direct
*trnN*	Asn	GTT	72	inverted
*trnW*	Trp	CCA	74	direct
*trnS*	Ser	GGA	87	inverted
mitochondrial origin				
*trnfM*	Met	CAT	74	inverted
*trnG*	Gly	GCC	72	direct
*trnI*	Ile	CAU	81	inverted
*trnK*	Lys	TTT	73	inverted
*trnQ*	Gln	TTG	72	direct
*trnS*	Ser	TGA	87	direct
*trnS*	Ser	GCT	88	direct
*trnY*	Tyr	GTA	83	inverted
*trnC*	Cys	GCA	71	inverted
*trnE*	Glu	TTC	72	inverted
*trnP*	Pro	TGG	75	inverted

Using ORF-Finder and BLAST searching, 50 ORFs longer than 100 codons were identified in the *E. sativa* mitochondrial genome. Among the 50 ORFs, only the *orf112*, *orf121*, *orf122*, and *orf275* have two copies. All others are single-copy ORFs. Most of the ORFs are between 300 and 500 bp in length, except for 10 ORFs that are longer than 500 bp, including the 1 200 bp *orf399* and the 1 911 bp *orf636*.

### Subgenomic circles mediated by large repeats

Large repeats (>1 Kb) have been identified in most of the seed plants analyzed, except for white mustard (*Brassica hirta*) (Palmer and Herbo, 1987). The repeats in the *E. sativa* mitochondrial genome were analyzed. Three pairs of large repeats were identified, accounting for 13.48% of the genome. The large repeats were designated as R1, R2 and R3 (Table 3). R1 (10 320 bp) has a pair of large repeats in the opposite orientation, while R2 (4 864 bp) and R3 (1 513 bp) have a pair of large repeats in the same orientation. Large repeat R1 contains two ORFs, *orf112* and *orf122*, while R2 and R3 contain *orf275* and the *orf121*, respectively. No known protein coding gene was found in these large repeats.

**Table 3:** Large repeats in the mtDNA of *Eruca sativa.*

No.	Type^a^	Size(bp)	Copy-1	Copy-2	Difference between copies	Identity
R1	IR	10320	77495-87814	176149-186468	identical	100%
R2	DR	4864	4083-8946	119763-124626	2 bp mismatch	99.95%
R3	DR	1513	1-1513	118250-119762	identical	100%

a DR and IR: direct and reverse repeats, respectively.

**Figure 2:**
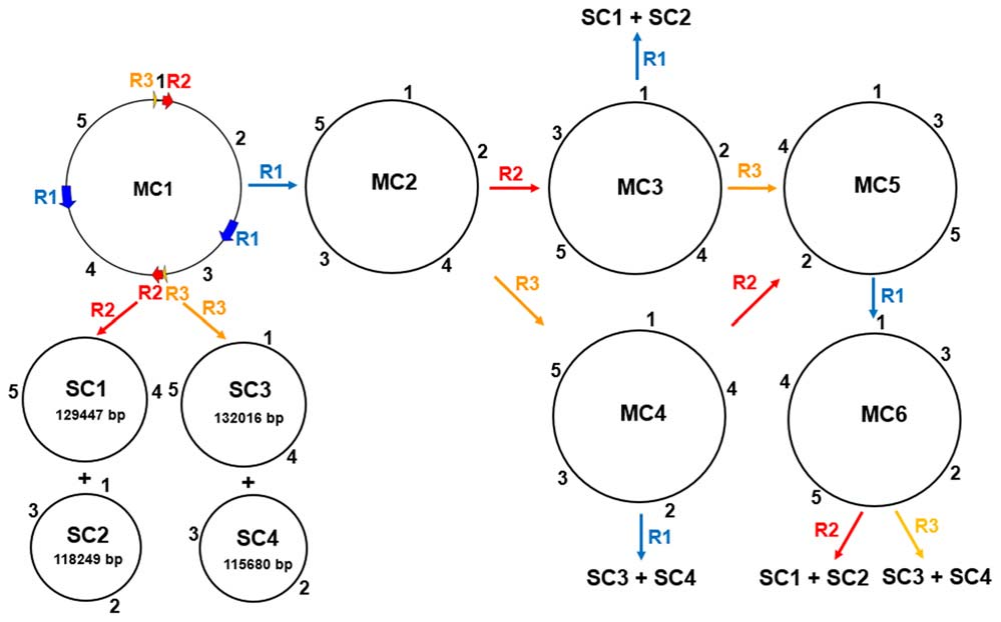
The multipartite mitochondrial genome structure of *Eruca sativa.* Schematic diagrams showing six master circles and four subgenomic circles. The three repeat pairs are shown in different colors. MC and SC mean master and subgenomic circles, respectively. Numbers outside circles indicate segments separated by repeat pairs.

Large repeats have been implicated in mediating high frequency, reciprocal DNA exchange that can result in subdivision of the genome into a multipartite configuration [31]. The formation of the multipartite structure of the *E. sativa* mitochondrial genome was predicted based on the assumptions of intra-molecular homologous recombination (Figure 2). Six isometric master circular (MC) genomic structures of the same length (including MC1 shown in Figure 1) could be produced by intra-molecular recombination between different repeat pairs. In addition, MC molecule 1 and 6 may generate four subgenomic circles, including two small circles of 129 447 bp (SC1) and 118 249 bp (SC2) via the pairwise large repeat R2, and another two small circles of 132 016 bp (SC3) and 115 680 bp (SC4) mediated by the pairwise large repeat R3. MC3 may produce SC1 and SC2, and MC4 may produce SC3 and SC4, mediated by the pairwise large repeat R1.

### Sequence comparison between *E. sativa* and *B. napus* mtDNAs

We compared the sequences of the mtDNAs from *E. sativa* and *B. napus*. Most of the protein coding and RNA genes were conserved in length, except *ccmFN2* and *rrn18*. The 5′ portion of the coding region of *ccmFN2* in *E. sativa* mtDNA was quite different (Figure S1) and a 25-bp deletion in *rrn18* was found in *E. sativa* mtDNA (Figure S2) compared with that in *B. napus*. The *E. sativa* mitotype is devoid of *cox2-2*, compared with that of *B. napus*. 27 single nucleotide polymorphisms (SNPs) were detected in 14 genes when compared with *B. napus* (Table 4). Thirteen synonymous substitutions were found in *atp6*, *ccmB*, *cob*, *cox1*, *nad2*, *nad6*, *rpl2*, *rps3*, and *rps4*. Fourteen nonsynonymous mutants were found in 11 genes, including an S to N (199aa) switch in *atp1*, a V to I (18aa) and an H to F (51aa) switch in *atp6*, a P to L (107aa) switch in *ccmB*, an R to K (113aa) switch in *ccmFC*, an H to Y (285aa) switch in *cob*, a P to L (112aa) switch in *cox1*, an S to L (126aa) and an S to N (438aa) switch in *matR*, a C to R (72aa) switch in *nad2*, an S to L (29aa) switch in *rpl2*, an L to P (172aa) switch in *rpl5*, and an M to I (50aa) switch in *rps7*. Of these 27 SNPs, most were transitions and only three were transversion (G→T in *nad2*, T→A in *cox1*, and T→A in *atp6*). All tRNAs in the *B. napus* mitochondrial genome were detected in *E. sativa* mtDNA. However, the ORFs were quite different between these two mitotypes.

**Table 4:** SNP in protein-coding genes of mtDNA between *Eruca sativa* and *Brassica napus.*

Gene	Position from the start codon	nucleotide variation		Position from the first amino acid	amino acid change
		*B.napus*	*E.sativa*		
*atp1*	596	AGT	AAT	199	S→N
*atp6*	7	GAG	AAG	260	Synonymous
	559	GAA	AAA	76	Synonymous
	635	ATG	AAA	51	H→F
	735	GAC	GAT	18	V→I
*ccmB*	235	CCC	TCC	129	Synonymous
	303	AAG	AAA	107	P→L
	577	GGG	AGG	15	Synonymous
*ccmFC exonB*	337	GCG	ACG	113	R→K
*cob*	330	ATG	ATA	285	H→Y
	409	TCC	CCC	258	Synonymous
*cox1*	335	CCC	CTC	112	P→L
	702	TAC	TAT	234	Synonymous
	1466	CCT	CCA	489	Synonymous
*matR*	377	CGG	TGG	126	S→L
	1313	AGC	AAC	438	S→N
	1566	GGG	GGA	522	Synonymous
*nad2 exonB*	179	CAA	CGA	72	C→R
*nad2 exonD*	500	GAT	TAT	25	Synonymous
*nad6*	388	CGC	TGC	77	Synonymous
*rpl2 exonB*	47	CGA	CAA	29	S→L
*rpl5*	44	CAG	CGG	172	L→P
*rps3 exonB*	685	GGT	AGT	302	Synonymous
	823	CTT	TTT	256	Synonymous
*rps4*	391	CTT	TTT	233	Synonymous
*rps7*	298	CAT	TAT	50	M→I

### Evolutionary relationships of the cruciferous mitotypes

To further illustrate the evolution of mitochondrial genomes within the Cruciferae family, the *E. sativa* mtDNA and other reported Cruciferous mtDNAs were compared using BLASTN [32]. *E. sativa* mtDNA was used as the reference sequence and similar regions in two or more mtDNA sequences were aligned. The alignable *E. sativa* sequence (93%) was 81% identical to that of *R. sativus* mtDNA. The sequence identity shared by the mtDNA of *E. sativa* and *Brassica* was more than 83%, with a coverage in the range of 83–85%. Only 63% of the *E. sativa* mtDNA matched those of *Arabidopsis thaliana*, with an identity of more than 68%, and the longest fragment was only 8.0 kb. This result suggested that the evolutionary relationship of mitochondrial genomes among *E. sativa*, the *Brassicas* and *R. sativus* is closer than that between *E. sativa* and *A. thaliana*.

**Figure 3:**
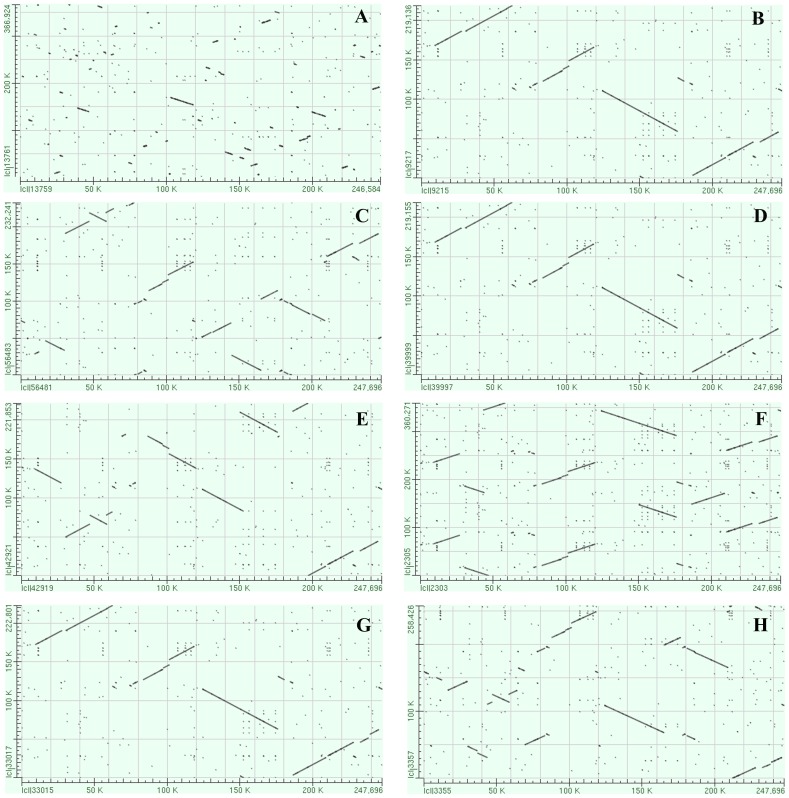
Dot matrix alignments of the *Eruca sativa* (x-axis) and other known cruciferous mtDNAs (y-axis). (A) *Arabidopsis thaliana* (*tha*), (B) *B. rapa* (*cam*), (C) *B. carinata* (*car*), (D) *B. juncea* (*jun*), (E) *B. napus* (*nap*), (F) *B. oleracea* (*ole*), (G) *B. napus* (*pol*), (G) *Raphanus sativus* (*sat*).

**Figure 4:**
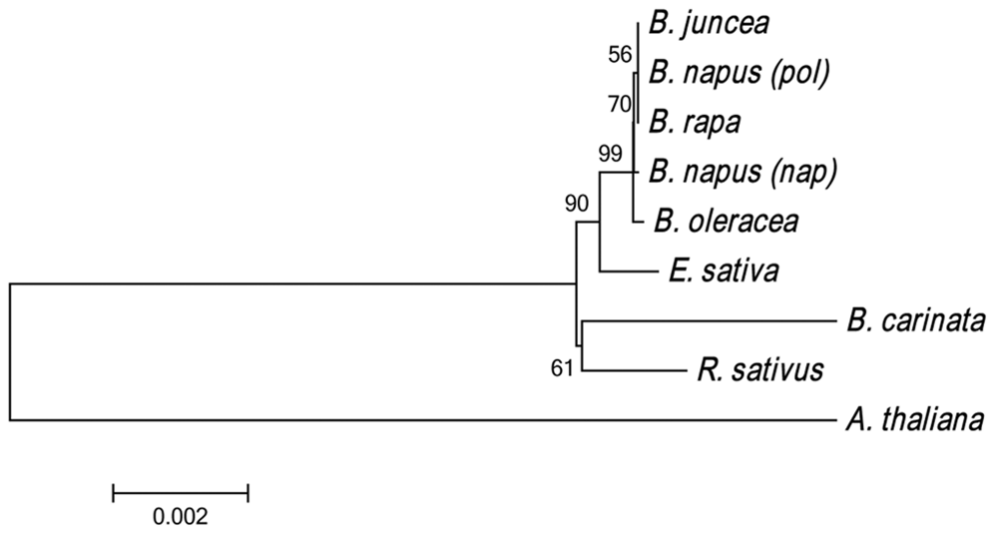
Phylogeny of nine cruciferous mitotypes. The phylogenetic tree was inferred using the neighbor-joining method based on the exons of 32 protein coding genes. The evolutionary distances were computed using the Kumar method, and the branch lengths are in units of synonymous substitutions per synonymous site. Evolutionary analyses were conducted in MEGA5.

In support of this hypothesis, a dot matrix analysis showed that the lengths of syntenic regions between *E. sativa* and *A. thaliana* are shorter than those between *E. sativa* and *Brassica* or *R. sativus*. Additionally, the distribution of syntenic regions between the mtDNAs of *E. sativa* and *A. thaliana* is more dispersed, and the identity is lower, than that between *E. sativa* and the *Brassica* mitotypes (Figure 3). Moreover, the phylogenetic relationships among the Cruciferae family (Figure 4) were inferred using the neighbor-joining method and 23 conserved genes among the reported Cruciferae mitotypes. The results are mainly consistent with previous reports based on mitochondrial genome analysis [22] and strongly support the conclusion that *E. sativa* is more closely related to the *Brassica* species and *R. sativus* than to *A. thaliana*.

## Discussion

The *Cruciferae* family is one of the largest dicot families of the flowering plant kingdom and includes several vegetable and oilseed crops, as well as several model species of great scientific, economic and agronomic importance [33]. Annotations for mitochondrial genomes from closely related species would improve the understanding of molecular evolution and phylogenetic relationships [34] in the *Cruciferae* family. *E. sativa*, a member of the Cruciferae family, is a conventional crop consumed as food and fodder. The economic potential of *E. sativa* lies in various other aspects, including the protein sources for edible purposes, a potential source of industrial oil, an effective biological control of crop pests and traditional pharmacopoeia for various purposes [35]. To better understand this important crop, the mitochondrial genome of *E. sativa* was sequenced and annotated.

*Cruciferae* mitochondrial genomes are generally small (208–367 kb) compared with other seed plants. The *E. sativa* mt genome (248 kb) is larger than most *Brassica* mitotypes, but smaller than that of *B. oleracea* (360 kb) *and A. thaliana* (367 kb). Comparison of the *E. sativa* mtDNA with the *B. napus* mtDNA revealed that the *cox2-2* gene was absent from the *E. sativa* mt genome. This gene was also absent from the genomes of *B. oleracea*, *B. carinata*, and Ogura-cms-cybrid (oguC) rapeseed mitotypes [25], [36]. A distinguishing feature of Cruciferae mitochondrial genomes is that the *ccmFN* genes are divided into two reading frames (*ccmFN1* and *ccmFN2*) [23]. The translation of *ccmFN2* has been confirmed in *A. thaliana* mitochondria, which demonstrated that *ccmFN2* was not a pseudo gene, although it lacks a classical ATG initiation codon [37]. Sequence alignments of *ccmFN2* from reported Cruciferae mtDNAs showed that the first 45 bp of the putative *ccmFN2* gene in *E. sativa* mt genome is quite different from the *ccmFN2* gene in *Brassica* and *A. thaliana* mitotypes (Figure S1), suggesting that this non-conserved region may not be critical for gene function. However, the tryptophan-rich WWD domain in *ccmFN2*, which is responsible for heme binding [38], is conserved among these mitotypes.

The 5S and 18S rRNA genes in *E. sativa* mtDNA are closely linked, as they are in other plants, and the 26S rRNA gene is separated from the 18S and 5S by 26 459 bp. To elucidate the evolutionary origins of mitochondria, the ribosomal RNA genes have been extensively examined [39]. Sequence analysis of the *rrn18* gene from wheat, maize and soybean showed highly similarity between the plant mitochondrial *rrn18* genes and the eubacterial 16S rRNA, suggesting that there is a much slower rate of sequence change in plant mitochondria compared with their animal counterparts [40]. We compared the *rrn18* among the reported *Cruciferae* mitotypes and found a 25-bp deletion in *rrn18* in E. sativa mtDNA (Figure S2) compared with that in *Brassica* mitotypes. We also noticed a 46-bp deletion in *rrn18* within the same region of the *Brassica* mitotypes when compared with that in *A. thaliana* mtDNA. However, the overall nucleotide identities of the *rrn18* gene sequences were markedly high, from 89.50% between *E. sativa* and *A. thaliana* to 93.92% between *E. sativa* and the *Brassica* family. The nucleotide identity of the *rrn18* gene between *E. sativa* and *R. sativus* was 93.86% (Figure S2). This result is consistent with the results of the phylogenetic analysis based on 32 protein coding genes (Figure 4), which suggested that *E. sativa* is closer to *Brassica* and *R. sativus* than to *A. thaliana*.

18 tRNA genes were identified in *E. sativa* mtDNA, accounting for only 0.56% of the mitochondrial genome. Among them, six seem to be chloroplast derived, which exhibit high sequence identity (>99%) to their chloroplast counterparts. The chloroplast-derived *trnH-GTG*, *trnM-CAT*, trnS-*GGA*, *trnW-CCA*, *trnD-GUC*, and *trnN-GTT* genes, which are frequently found in mitochondrial genomes of angiosperms [15], were identified in the *E. sativa* mtDNA. An additional chloroplast-originating tRNA gene (*trnL-CAA*), which is found in the *R. sativus* and *Brassica* mitotypes [22], was also identified in *E. sativa* mitochondrial genome. This result indicated that mt tRNA genes are frequently transferred from chloroplast genomes during the evolution of angiosperms. However, another two gene (*trnP-GGG* and *trnQ-UUG*) transfer events reported in dicots [41], [42] were not found in *E. sativa.*

Genes with known functions are relatively conserved among the *Cruciferae* mitotypes, especially for the protein coding genes. However, the mitochondrial genomes structural differences are remarkable among the *Cruciferae* family. Multipartite structures of mtDNA mediated by large repeats have been commonly observed in plant species [43]. Direct electron-microscopic evidence of the coexistence of multipartite molecules in the plant mitochondrial genome has been found in tobacco [44]. The large repeat, RB, which is 2,427 bp in length and has been identified in most of the reported *Brassica* (except the oguC rapeseed) mitotypes, was not found in the *E. sativa* mtDNA. Instead, three pairwise large repeats were identified. Large repeat R1 in *E. sativa* mtDNA showed significantly high sequence similarity to the 6 580-bp large repeat R in *B. carinata* mitochondria (Figure S3). The 1 513-bp large repeat R3 showed 99% identity to the corresponding segments of the large repeat R2 in *B. oleracea* mitochondrial genome. Only 2% and 23% of R1 in *E. sativa* mtDNA showed high similarity (>83%) with the large repeats in *A. thaliana* and *R. sativus* mtDNA, respectively. The tripartite structure of the mitochondrial genome, including one master circle and two smaller subgenomic circles, has been reported in *Brassica* species (except the *ole* mitotype) and *R. sativus* [22], [25], [36]. The predicted multipartite structure of the mitochondrial genome in *E. sativa* is more complex than other *Cruciferae* species because of the three pairwise large repeats, including six master circles and four smaller subgenomic circles.

## Conclusions

In this study, we reported the complete mitochondrial genome sequence of *E. sativa*, a member of the *Cruciferae* family. The *E. sativa* mtDNA is 247 696 bp and harbors 33 known protein coding genes, three rRNAs (5 S, 18 S, and 26 S rRNAs) and 18 tRNAs. In addition, the *cox2-2* gene is absent, the *ccmFN2* and *rrn18* genes have different lengths and 27 SNPs are involved in 14 protein coding genes in comparison with *B. napus* mtDNA. Reorganization of the genome may have occurred via three pairs of large repeats, resulting in a more dynamic structure of the *E. sativa* mtDNA compared with other cruciferous mitotypes. This may produce six master circles and four smaller subgenomic circles. The evolutionary relationships analysis among reported *Cruciferous* mitotypes revealed that the mitochondrial genome of *E. sativa* is divergent from *A. thaliana*, but closely related to those of *Brassica* and *R. sativus*. This study will improve our understanding of the *E. sativa* crop and the evolution of mitochondrial genomes within the *Cruciferae* family.

## Supporting Information

Figure S1 **Sequence alignments of** ***ccmFN2*** **from reported Cruciferae mtDNAs.** The highly and partly conserved amino acids are shaded black or grey respectively. The black block diagram indicates the un-conserved region of *ccmFN2* in *E. sativa* mtDNAs compared to other reported Cruciferae mtDNAs.
(TIF)

Figure S2 **Sequence alignments of** ***rrn18*** **from reported Cruciferae mtDNAs.** The highly and partly conserved amino acids are shaded black or grey respectively. The black block diagram indicates the deletion region of *rrn18* in *E. sativa* mtDNAs compared to other reported Cruciferae mtDNAs.
(TIF)

Figure S3 **Alignment of the large repeats in** ***Eruca sativa*** **mtDNA with the large 6.6 kb repeats in** ***car*** **.** The alignment was made using Mauve. Blocks of the same color denote homologous regions; the *B. carinata* blocks above or below the middle line represent direct or inverted, respectively, compared with *E. sativa*. The extent to which a block is filled indicates the similarity of the syntenic region.
(TIF)
