# Supplementary figures and images for: Complete Mitochondrial Genome of Eruca sativa Mill. (Garden Rocket)
Source: PLoS One. 2014 Aug 26;9(8):e105748. doi: 10.1371/journal.pone.0105748 (PMC4144905; doi:10.1371/journal.pone.0105748)

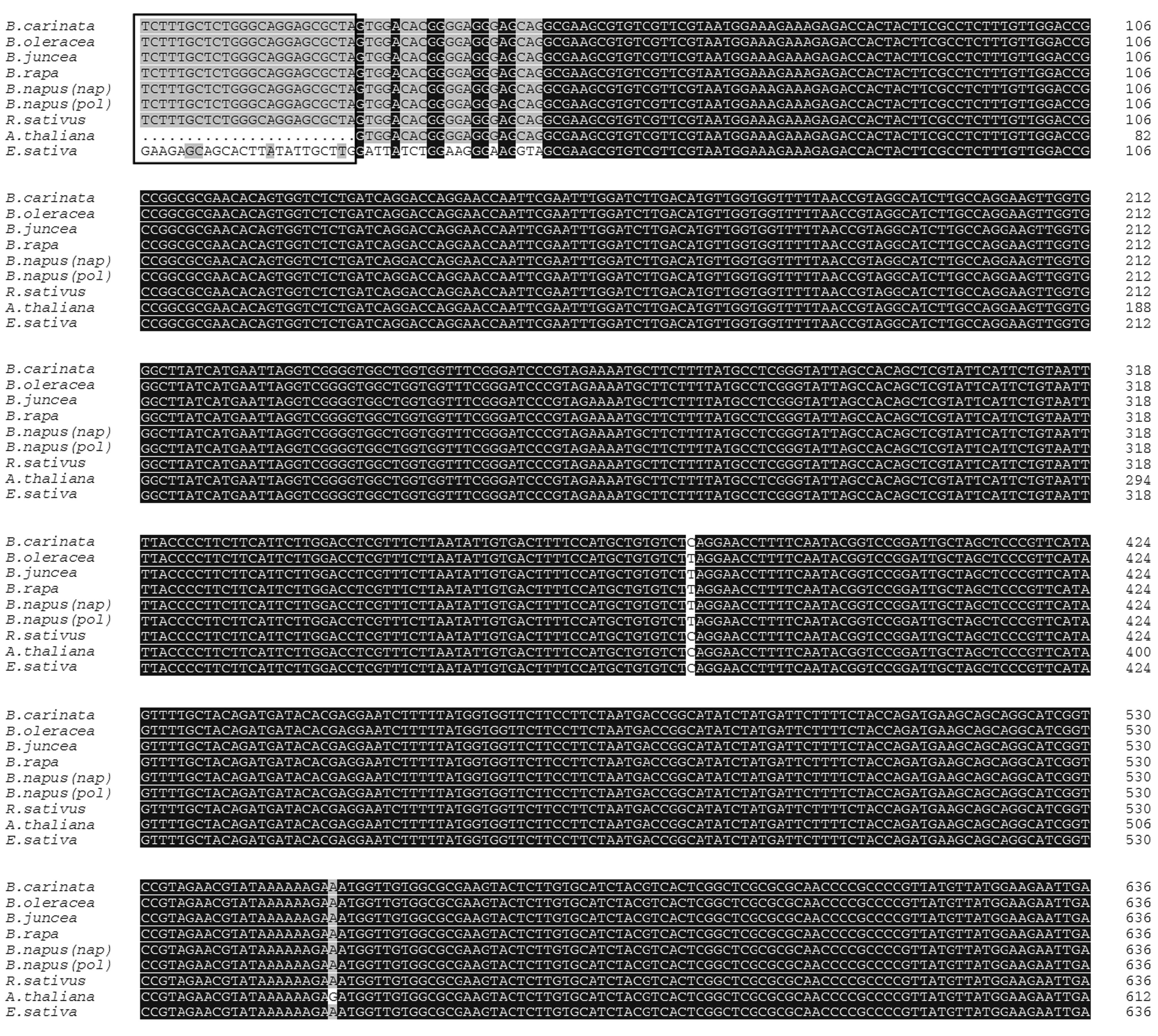

Supplement: Figure S1 — Sequence alignments of ccmFN2 from reported Cruciferae mtDNAs. The highly and partly conserved amino acids are shaded black or grey respectively. The black block diagram indicates the un-conserved region of ccmFN2 in E. sativa mtDNAs compared to other reported Cruciferae mtDNAs. (TIF) [file pone.0105748.s001.tif]

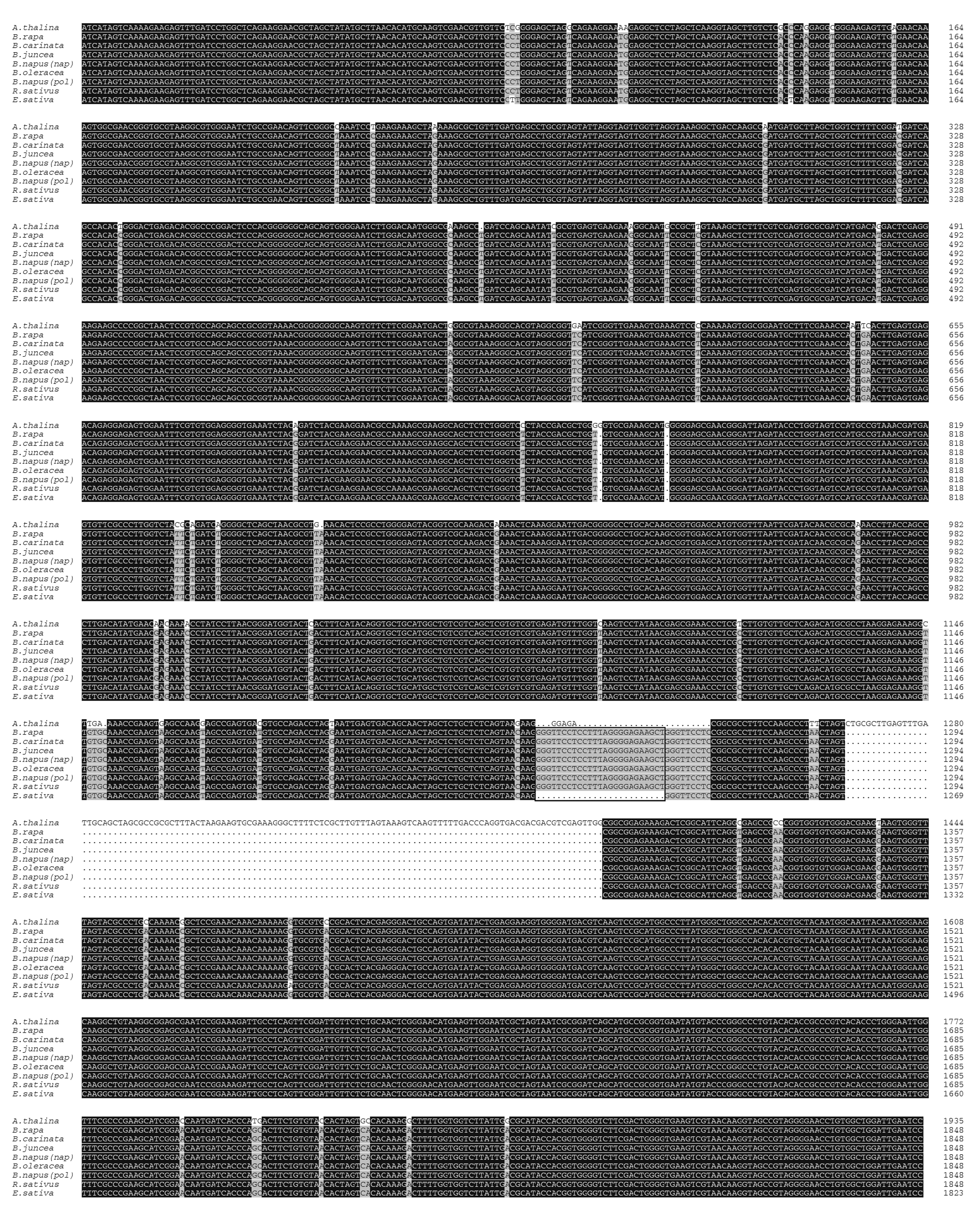

Supplement: Figure S2 — Sequence alignments of rrn18 from reported Cruciferae mtDNAs. The highly and partly conserved amino acids are shaded black or grey respectively. The black block diagram indicates the deletion region of rrn18 in E. sativa mtDNAs compared to other reported Cruciferae mtDNAs. (TIF) [file pone.0105748.s002.tif]

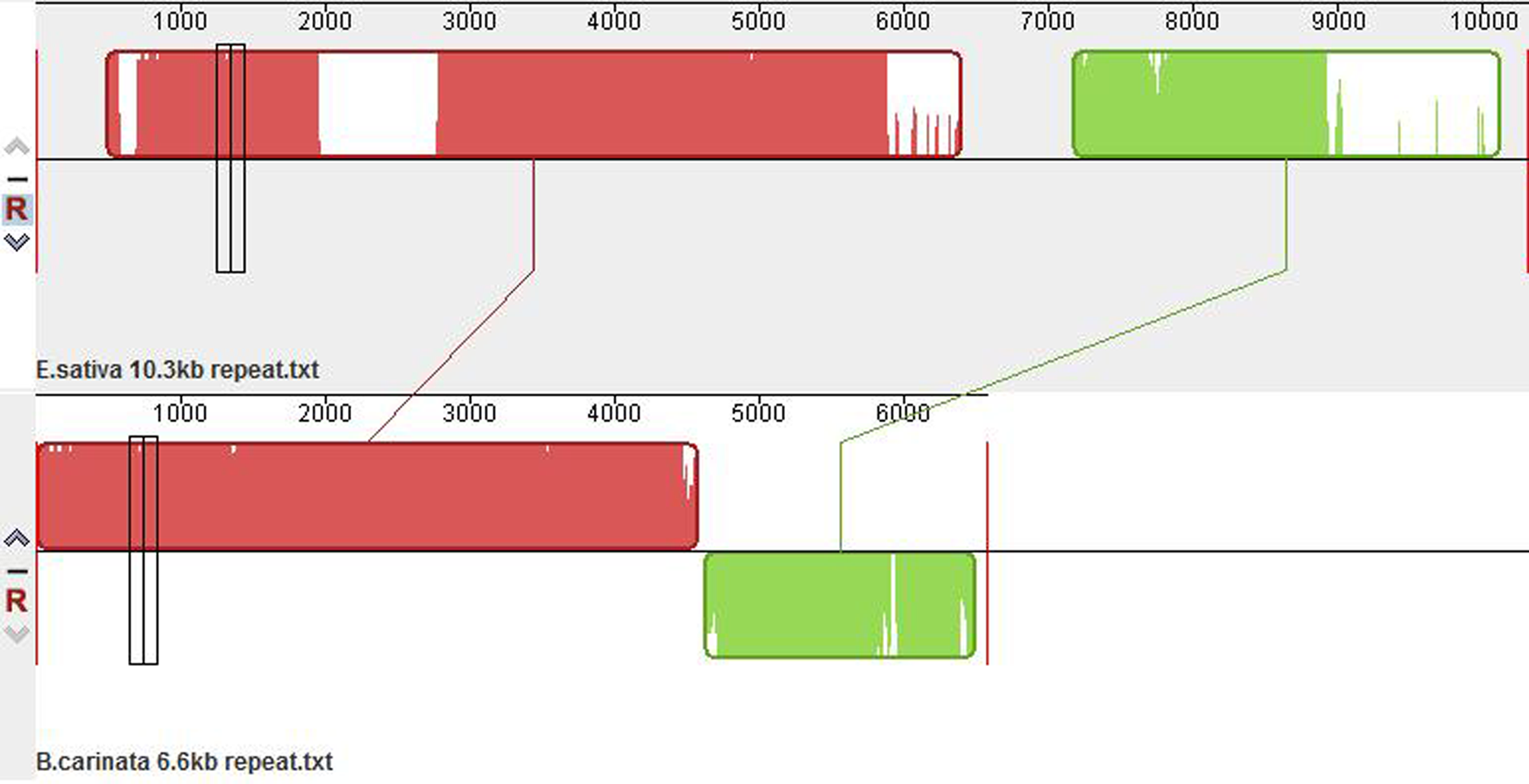

Supplement: Figure S3 — Alignment of the large repeats in Eruca sativa mtDNA with the large 6.6 kb repeats in car . The alignment was made using Mauve. Blocks of the same color denote homologous regions; the B. carinata blocks above or below the middle line represent direct or inverted, respectively, compared with E. sativa. The extent to which a block is filled indicates the similarity of the syntenic region. (TIF) [file pone.0105748.s003.tif]
